# Near-infrared spectroscopy for kidney oxygen monitoring in a porcine model of hemorrhagic shock, hemodilution, and REBOA

**DOI:** 10.1038/s41598-024-51886-y

**Published:** 2024-02-01

**Authors:** Natalie A. Silverton, Lars R. Lofgren, Kai Kuck, Gregory J. Stoddard, Russel Johnson, Ali Ramezani, Guillaume L. Hoareau

**Affiliations:** 1 Department of Anesthesiology, University of Utah, Helix Building 5050, 30N Mario Capecchi Dr., Salt Lake City, UT 84132 USA; 2 Geriatric Research, Education and Clinical Center, VA Medical Center, 500 Foothill Dr, Salt Lake City, UT 84148 USA; 3 Division of Epidemiology, Department of Internal Medicine, University of Utah, 295 Chipeta Way, Rm 1N433, Salt Lake City, UT 84132 USA; 4 Department of Emergency Medicine, Helix Building 5050, 30 N Mario Capecchi, Room 2S240, Level 2, South, Salt Lake City, UT 84132 USA; 5 Department of Emergency Medicine, Nora Eccles Harrison Cardiovascular Research and Training Institute, Helix Building 5050, 30 N Mario Capecchi, Room 2S240, Level 2, South, Salt Lake City, UT 84132 USA

**Keywords:** Acute kidney injury, Trauma, Biomedical engineering

## Abstract

Acute kidney injury is a common complication of trauma and hemorrhagic shock. In a porcine model of hemorrhagic shock, resuscitative endovascular balloon aortic occlusion (REBOA) and hemodilution, we hypothesized that invasive kidney oxygen concentration measurements would correlate more strongly with noninvasive near infra-red spectroscopy (NIRS) oxygen saturation measurements when cutaneous sensors were placed over the kidney under ultrasound guidance compared to placement over the thigh muscle and subcutaneous tissue. Eight anesthetized swine underwent hemorrhagic shock 4 of which were resuscitated with intravenous fluids prior to the return of shed blood (Hemodilution protocol) and 4 of which underwent REBOA prior to resuscitation and return of shed blood (REBOA protocol). There was a moderate correlation between the NIRS and kidney tissue oxygen measurements (*r* = 0.61 *p* < 0.001; *r* = 0.67 *p* < 0.001; *r* = 0.66 *p* < 0.001for left kidney, right kidney, and thigh NIRS respectively). When the animals were separated by protocol, the Hemodilution group showed a weak or nonsignificant correlation between NIRS and kidney tissue oxygen measurements (*r* = 0.10 *p* < 0.001; *r* = 0.01 *p* = 0.1007; *r* = 0.28 *p* < 0.001 for left kidney, right kidney, and thigh NIRS respectively). This contrasts with the REBOA group, where left and right kidney as well as thigh NIRS were moderately correlated with kidney tissue oxygen (*r* = 0.71 *p* < 0.001; *r* = 0.74 *p* < 0.001; *r* = 0.70 *p* < 0.001; for left kidney, right kidney, and thigh NIRS respectively). There was a strong correlation between both kidney NIRS signals and thigh NIRS measurements (*r* = 0.85 *p* < 0.001; *r* = 0.88 *p* < 0.001;for left kidney vs thigh and right kidney vs thigh respectively). There was also a strong correlation between left and right kidney NIRS (*r* = 0.90 *p* < 0.001). These relationships were maintained regardless of the resuscitation protocol. These results suggest that kidney NIRS measurements were more closely related to thigh NIRS measurements than invasive kidney tissue oxygen concentration.

## Introduction

Trauma is the leading cause of death for Americans under the age of 46 and is the third leading cause across all ages, accounting for approximately 100,000 deaths per year^1,2^. Hemorrhage is responsible for 30–40% of trauma mortality in the United States^3^. Hemorrhage that does not immediately lead to the death of a trauma victim, however, often results in acute kidney injury (AKI). This complication occurs in 43% of critically injured individuals and is associated with significant increases in mortality^4^. Reduced oxygen delivery to the kidneys secondary to bleeding and hypovolemia is the cornerstone of AKI after critical injury.

Resuscitative Endovascular Balloon Aortic Occlusion (REBOA) is a potential therapy to treat patients with severe injuries and profound blood loss due to non-compressible torso hemorrhage**.** A balloon-tipped catheter is inserted through the femoral artery and positioned in the aorta. The balloon can then be inflated to maintain blood flow to the major organs of the upper body, such as the heart and brain, at the expense of blood flow to other organs below the balloon, such as the kidneys. While a life-saving measure, the impact of prolonged balloon inflation on kidney hypoxia may be profound. REBOA also provides a unique opportunity to provide a translational model of survivable and profound renal ischemia-injury with direct clinical correlates. A real-time bedside monitor of kidney oxygenation during hemorrhage and REBOA might improve the care of critically ill trauma patients by allowing for early diagnosis of kidney hypoxia.

Near-infrared spectroscopy (NIRS) is a noninvasive technology used to determine regional tissue oxygen saturation. NIRS relies on a sensor attached to the skin that emits light in the near-infrared spectrum (700–900 nm). This wavelength of light can penetrate underlying tissues and is within the range of absorption of oxygenated and deoxygenated hemoglobin. While pulse oximetry measures arterial hemoglobin saturation, NIRS measures a mixture of arterial and venous capillary oxygenation, making it well-suited to determine tissue oxygen delivery and perfusion^5^. NIRS is routinely used to measure the capillary oxygen saturation of the brain's frontal lobe when patients are undergoing cardiac or vascular surgery. The NIRS sensor consists of a single light emitter and two light sensors at various distances from the emitter. The depth of penetration of NIRS is related to the distance from the light emitter to the detector. Subtracting the closer detector signal from that of the far detector, in theory, allows for measurements that are specific to the underlying brain tissue rather than the superficial skin or the skull^6,7^. Cerebral oximetry measured with NIRS correlates with jugular venous oxygen saturation suggesting that this noninvasive technology reflects tissue oxygenation^8^. A large body of literature describes the use of NIRS in neonates and infants both as a systemic perfusion monitor and a regional monitor of kidney perfusion^5,9–13^. The maximum depth of penetration of NIRS, however, is thought to be only 2.5 cm, thereby limiting the use of NIRS as a kidney perfusion monitor in adults^14–16^. Recently, however, two studies in adult cardiac surgery patients using ultrasound guided placement of NIRS sensors over the kidney found that intraoperative regional tissue oxygen desaturations were associated with the subsequent development of AKI^17,18^. A third study found a correlation between NIRS oxygen saturation measurements over the kidney and renal venous oxygen saturation in adult cardiac surgery patients^19^. In all three studies, patients were excluded for body mass index > 30 kg/m^2^ or if the surface of their kidney was > 4 cm from the skin.

We have developed a porcine model of hemorrhage and REBOA in which we measure kidney oxygen concentration directly by placing an oxygen sensor in the renal medulla^20^. Using this oxygen sensor, we can detect renal hypoxia during the hemodynamic changes that occur with hemorrhage and REBOA. We designed the current study to determine whether invasive kidney tissue oxygen concentration correlates with noninvasive NIRS oxygen saturation when these sensors are placed on the skin over the kidney using ultrasound guidance. REBOA creates a profound ischemic condition, leading to severe vasodilation and hemodynamic instability even after resuscitation with whole blood^21–23^. We, therefore, created a second model of hemorrhage and hemodilution to assess the relationship between invasive and noninvasive kidney oxygen monitoring in the setting of more subtle hemodynamic changes that may apply to a broader patient population. We hypothesized that measurements from NIRS sensors placed on the skin over the kidney would correlate more strongly with direct medullar kidney oxygen measurements than those obtained from NIRS sensors placed on the skin over the thigh. We also hypothesized that this relationship would hold true under both the extreme hemodynamic instability that occurs with REBOA and the less severe changes that occur with hemorrhage and hemodilution.

## Methods

### Overview

**Figure 1:**
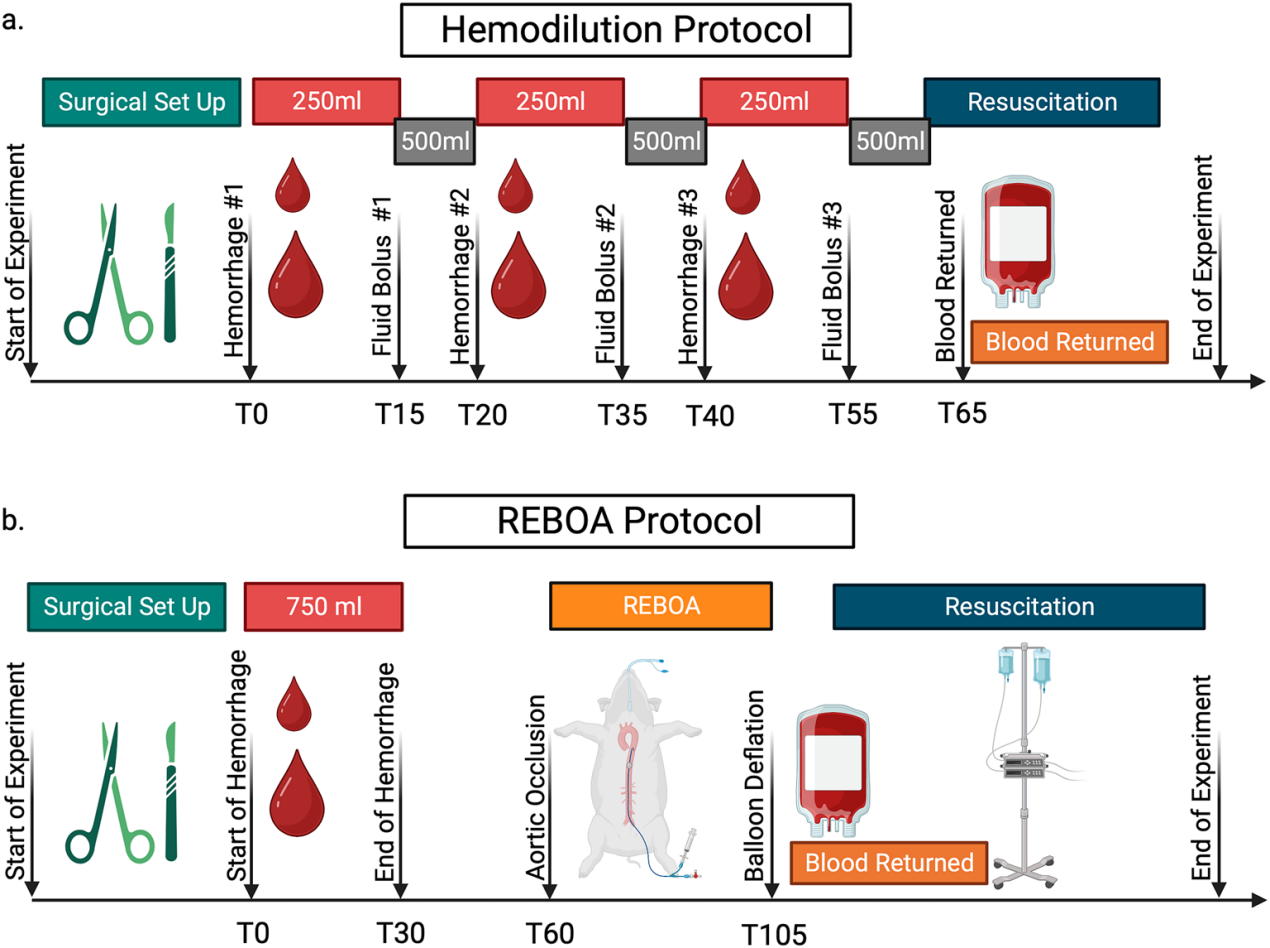
(**a**) Description of the Hemodilution protocol. Each animal underwent surgical instrumentation followed by 3 separate periods of hemorrhage where 250 ml of blood was removed from the brachial artery. Each hemorrhage period was followed a 500 ml bolus of balanced IV fluids. At the end of the 3 hemorrhage periods, all 750 ml of shed blood was returned to the animal. This was followed by a resuscitation period in which mean arterial pressure was maintained > 65 mmHg with fluid boluses and norepinephrine as needed. (**b**). Description of the REBOA protocol. Each animal underwent surgical instrumentation followed by a single hemorrhage period when 750 ml of blood was removed from the brachial artery. This was followed by a 30-min period of anemia. Resuscitative endovascular balloon occlusion of the aorta (REBOA) was then maintained for 45 min followed by gradual balloon deflation and simultaneous return of shed blood. This was followed by a resuscitation period in which mean arterial pressure was maintained > 65 mmHg with fluid boluses and norepinephrine as needed.

The animal research: reporting of in vivo experiments guidelines (ARRIVE) were used to prepare this manuscript. The Institutional Animal Care and Use Committee at the University of Utah approved this study (Protocol number 00001819; Approved 10/28/2021) and all experiments were performed in accordance with the Guide for the Care and Use of Laboratory Animals and the Animal Welfare Act Regulations and Standards. An overview of the experimental protocols can be seen in Fig. 1.

### Animal preparation

Ten castrate male Yorkshire swine or non-pregnant females weighing 53–60 kg and 6–8 months old were acclimatized in temperature- and light-controlled enclosures with given access to environmental enrichment for at least 7 days. Animals were fasted overnight but allowed free access to water prior to induction of anesthesia. Anesthesia was induced with a combined intramuscular injection of ketamine (2.2 mg/kg, Vedco, Saint Joseph, MO) and xylazine (2.2 mg/kg, Vedco, Saint Joseph, MO) and after endotracheal intubation, anesthesia was maintained at 1–2.5% isoflurane (Dechra, Northwich, United Kingdom) in 2 L/min of oxygen. Animals were mechanically ventilated with a positive end-expiratory pressure of 4 cm H_2_O and a fraction of inspired oxygen between 40 and 100% to maintain pulse oximetry > 95%. Tidal volumes of 6–8 ml/kg were used with the respiratory rate adjusted to maintain end-tidal CO_2_ of 35–45 mmHg. Balanced isotonic fluids (Plasmalyte 148, Baxter, Deerfield, IL) were administered at 5 ml/kg/hr intravenously as maintenance fluid. Ionized calcium, potassium, and glucose were monitored throughout the experiment and treated as needed. Warming blankets were used to maintain normothermia.

### Surgical preparation

Lidocaine was used for local infiltration. Percutaneous vascular access was established with ultrasound guidance and the Seldinger technique in the following locations. A 6 Fr carotid artery catheter was placed for upper body blood pressure monitoring. A 7 Fr brachial artery catheter was placed for controlled hemorrhage and blood sampling. A 12 Fr femoral artery catheter was inserted to introduce a custom-made 7 Fr REBOA catheter (Custom-made, Certus Critical Care). This femoral artery catheter was also used for lower body blood pressure monitoring. In 4 animals, a REBOA balloon was positioned immediately superior to the diaphragm. This placement was confirmed with fluoroscopy. A 9 Fr introducer catheter was placed in the internal jugular vein to deliver fluids and autologous blood and then a triple-lumen catheter was inserted through the sheath for central venous pressure monitoring and medication infusions. A 9 Fr introducer sheath was placed in a femoral vein for fluid bolus administration. A midline laparotomy was performed, and 20 Fr Foley catheter was placed in the bladder via a cystotomy. A splenectomy was performed to prevent auto-transfusion.

**Figure 2:**
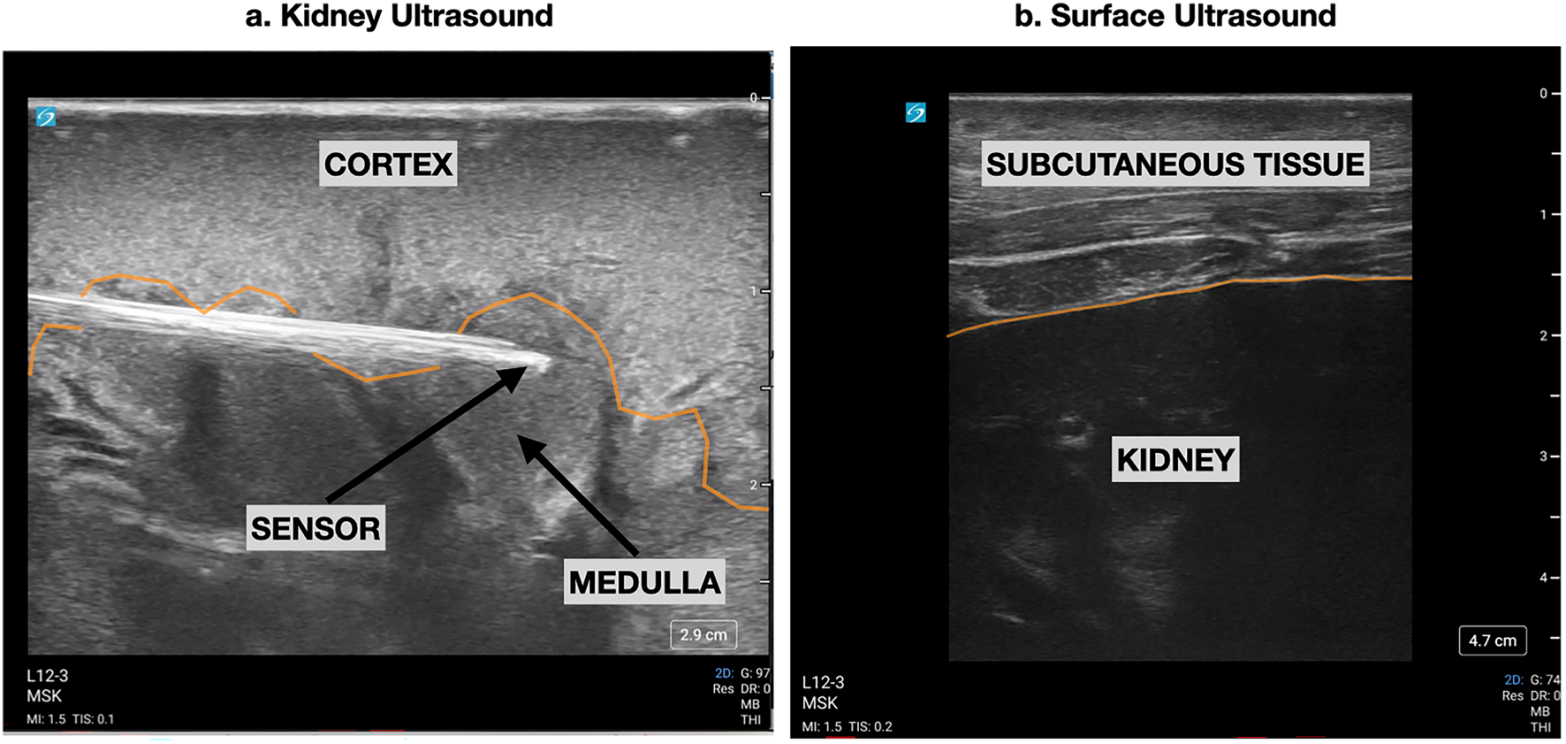
(**a**). Ultrasound image acquired by placing the linear transducer directly over surface of the left kidney. The 20 g IV can be seen in long axis with the tip of the oxygen sensor extending out into renal medullary tissue. (**b**). Ultrasound image acquired by placing the linear transducer over the skin of the left flank just below the last rib. Subcutaneous muscle and fat are seen overlying the surface of the kidney. This area was marked and a near infra-red spectroscopy sensor was placed on the skin in this location.

The left kidney was dissected, a 20 g IV catheter was then placed in the renal medulla under ultrasound guidance, and a fiberoptic probe was threaded through the catheter until it was visualized in the renal medulla (Fig. 2a). This probe is a luminescent oxygen sensor and a Doppler-based flow sensor designed for oxygen and perfusion monitoring (Oxford Optronix, Abington, UK). The probe and IV catheter were then glued in place (Vetbond, 3 M, St Paul, MN). The left kidney was then returned to the retroperitoneal space. The abdomen was then closed until the end of the experiment. The surgical set-up described for this model has been previously published by our group^20^.

Surface ultrasound was used to locate both kidneys, skin-to-kidney surface measurements were recorded, and NIRS sensors (Foresight, Edwards Lifesciences, Irvine, CA) were placed on the skin directly over the kidneys bilaterally (Fig. 2b). A NIRS sensor was also placed over the left thigh. These were Large ForeSight NIRS sensors (model number FSESL) and were connected to a Foresight Elite monitor (CAS Medical Systems, Branford, CT). The source-detector distances for these sensors are 15 mm and 50 mm for shallower and deeper tissue measurements respectively. Flank mode was used on the monitor for NIRS sensors placed on the skin over the left and right kidney and thigh mode was used for the NIRS sensors placed on the skin over the thigh.

### Hemorrhage and resuscitation protocols

Before starting the experimental procedure, there was a stabilization period of at least 10 min. During this time, mean arterial pressure (MAP) was maintained at > 65 mmHg. If below that threshold, 5/ml/kg boluses of balanced isotonic crystalloid were given. After 2 boluses, an infusion of norepinephrine (starting dose 0.02 mcg/kg/min) was titrated until target MAP was achieved. Animals were then randomly allocated into one of two hemorrhage and resuscitation protocols.

In the hemodilution protocol (Fig. 1a.), 250 ml of whole blood (approximately 7% of blood volume estimated as 60 mL/kg x body weight in kg) was removed from the brachial artery catheter over 15 min. Drawn blood was collected in citrated blood collection bags under constant agitation. Blood was stored in a warm water bath at 38 °C. At the end of each hemorrhage period, a 500 ml bolus of balanced isotonic crystalloid was given over 5 min. This procedure was repeated 3 times. After the third bolus of balanced isotonic crystalloid, all 750 ml (approximately 21% of blood volume) of shed blood were returned, and the animal was observed for 20–30 min.

In the REBOA protocol (Fig. 1b), all 750 ml of blood were removed over 30 min. Animals then remained untreated for an additional 30 min, after which they were then subjected to 45 min of REBOA. At the end of the REBOA period, the balloon was slowly deflated over 10 min as the animals were transfused their shed blood. A critical care period ensued during which animals were resuscitated with intravenous balanced crystalloids and vasopressors (norepinephrine) according to a pre-specified algorithm (Supplemental Fig. 1) for 255 min until the end of the experiment. At the end of each experiment, the animal was euthanized.

### Data collection

Upper body (carotid) and lower body (femoral) MAP were calculated from the continuous arterial blood pressure traces which were resampled to 1 Hz throughout the experiment. Renal medullary tissue oxygen concentration was recorded using the Oxford Optronix luminescent oxygen probe and Doppler blood flow sensor. Data from these sensors were sampled every second (PowerLab data acquisition platform, ADInstruments, Colorado Springs, CO). Regional tissue oxygen saturation using NIRS was recorded over the kidneys bilaterally and the left thigh (CAS Medical Systems, Branford, CT). Data from the NIRS sensors was sampled every 2 s.

### Serum analysis

Arterial whole blood was sampled at baseline, after each hemorrhage period in the Hemodilution protocol, and every 30 min in the REBOA protocol. Plasma lactate concentration, hemoglobin concentration, blood gases and electrolytes concentrations were measured at these intervals (iStat, Abbott, Chicago, IL).

### Statistics

Descriptive statistics were reported as mean (standard deviation). Mean arterial pressure data were collapsed to a single mean value for each animal for the three time periods reported (baseline, hemorrhage, and resuscitation). For the baseline and resuscitation characteristics, a Student’s *t*-test was used for continuous data and a Fisher exact test for categorical data. Our primary comparison was between direct kidney tissue oxygen concentration and the three NIRS measurements (left kidney, right kidney, and thigh). These data were compared using a “within subjects” correlation coefficient, which accounts for the lack of independence among repeated measures by removing the variation between subjects^24^. A mixed-effects linear regression was also used to model the relationship between NIRS oxygen saturation measurements and direct kidney medullary oxygen concentration. This also accounts for lack of independence of repeated measurements within the same animal. A Bland–Altman analysis for clustered data was performed for comparison of measurements with the same units in order to determine the mean difference and 95% limits of agreement. All significance tests were two-tailed, with *p* < 0.05 considered statistically significant. The analysis used STATA version 17.0 (Statcorp, College Station, TX).

## Results

**Figure 3:**
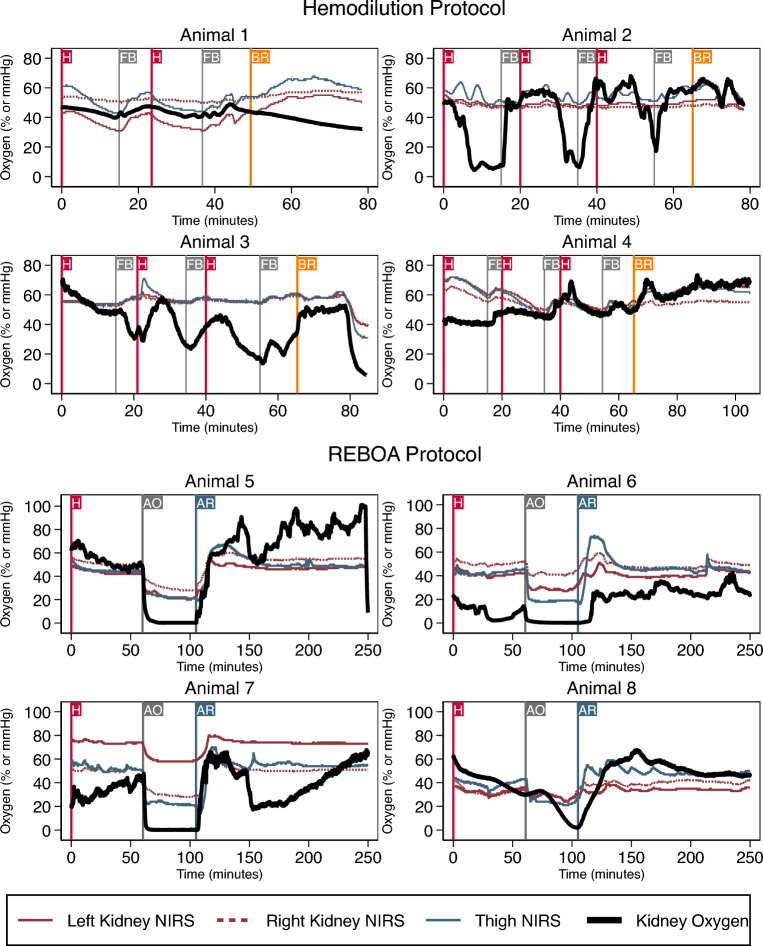
Left kidney, right kidney, and thigh near infra-red spectroscopy (NIRS) are compared to direct kidney tissue oxygen measurements over time for 4 animals undergoing the Hemodilution protocol. The red line marked “H” represents the start of each hemorrhage. The gray line marked “F” represents the end of each hemorrhage and the start of a bolus of balanced crystalloid. The orange line marked “BR” represents the time when shed blood was returned to the animal. For the resuscitative endovascular balloon aortic occlusion (REBOA) protocol, the red line marked “H” represents the start of the hemorrhage period. The gray line marked “AO” represents the start of aortic occlusion. The blue line marked “AR” represents the end of aortic occlusion when the aortic balloon was gradually released. For all graphs, the red solid and dashed lines indicate left and right kidney NIRS respectively and the blue solid line indicates thigh NIRS. The black solid line represents kidney tissue oxygen concentration. NIRS data were sampled every 2 s and kidney tissue oxygen was sampled every second.

In all animals, the surface of the kidney was between 1.5 and 2.0 cm from the skin. One animal in the REBOA protocol suffered a cardiac arrest immediately after surgical instrumentation and data could not be obtained. A second animal in the Hemodilution group was excluded from the analysis because the kidney oxygen sensor malfunctioned. The individual NIRS saturation and kidney oxygen concentration data for all 8 animals are presented in Fig. 3. One animal in the Hemodilution group only underwent two hemorrhage periods because the hemoglobin concentration decreased to 5.0 mg/dL after the second hemorrhage, and it was thought that a third hemorrhage might be fatal. Animals 1 and 4 in the hemodilution protocol appeared to have a temporal relationship between invasive kidney oxygen concentration and NIRS measurements. In animals 2 and 3 however, there appeared to be very little variation in NIRS measurements despite large changes in kidney oxygen concentration. In the REBOA protocol, animals 5, 6, and 7 had abrupt decreases in kidney oxygen concentration and NIRS measurements with balloon inflation and these were followed by increases in oxygenation after release of the balloon. In animal 8, kidney oxygen concentration initially remained elevated after balloon inflation. This was thought to be due to an incomplete occlusion of blood flow to the renal arteries rather than a measurement artifact. The aortic balloon was then hyperinflated and subsequently, kidney oxygen concentration decreased.

Table 1 summarizes the baseline and resuscitation data for the animals in each experimental protocol. Baseline characteristics were similar between the two groups. Peak plasma lactate concentration in the REBOA group was significantly higher than in the hemodilution group, otherwise resuscitation characteristics were also similar between the two groups.

**Table 1:** Baseline and resuscitation data. Hgb = hemoglobin; IQ*r* = interquartile range; kg = kilograms; MA*p* = mean arterial pressure; n = number of observations in the comparison; REBOA = resuscitative endovascular balloon aortic occlusion; SD = standard deviation.

	All Animals (8)	Hemodilution Protocol (4)	REBOA Protocol (4)	P
Weight (kg) Mean (SD)	56.4 (3.0)	57.2 (2.9)	55.7 (3.2)	0.530
Male n (%)	3 /8 (37.5)	2 /4 (50)	1/4 (25)	> 0.999
Baseline MAP (mmHg) Mean (SD)	85 (10)	82 (9)	89 9)	0.296
Baseline Hgb (mg/dL) Mean (SD)	10.1 (1.5)	10.4 (1.8)	9.6 (1.1)	0.508
MAP during hemorrhage (mmHg) Mean (SD)	68 (12)	70 8)	66 (15)	0.633
Nadir Hgb (mg/dL) Mean (SD)	6.6 (1.1)	5.9 (0.6)	7.3 (1.1)	0.058
MAP after return of blood (mmHg) Mean (SD)	82 (14)	90 (17)	74 (5)	0.121
Peak Lactate (mmol/L) Mean (SD)	7.7 (4.7)	3.5 (1.8)	11.8 (0.6)	< **0.001**

Significant values are in [bold].

The relationship between NIRS oxygen saturations and direct kidney tissue oxygen measurements is shown in Fig. 3. Pooled data from all 8 animals in both groups showed a moderate correlation between the kidney NIRS and kidney tissue oxygen measurements (*r* = 0.61 *p* < 0.001; *r* = 0.67 *p* < 0.001; for left and right kidney NIRS respectively). The correlation between thigh NIRS and kidney tissue oxygen, however, was similar (*r* = 0.66 *p* < 0.001). When the animals were separated by protocol, the Hemodilution group showed a weak or nonsignificant correlation between left or right kidney NIRS and kidney tissue oxygen measurements (*r* = 0.10 *p* < 0.001; *r* = 0.01 *p* = 0.1007; left and right kidney NIRS respectively). The correlation between thigh NIRS and kidney tissue oxygen was also poor (*r* = 0.28 *p* < 0.001). This contrasts with the REBOA group, where left and right kidney as well as thigh NIRS were moderately correlated with kidney tissue oxygen (*r* = 0.71 *p* < 0.001; *r* = 0.74 *p* < 0.001; *r* = 0.70 *p* < 0.001; for left kidney, right kidney, and thigh NIRS respectively).

**Figure 4:**
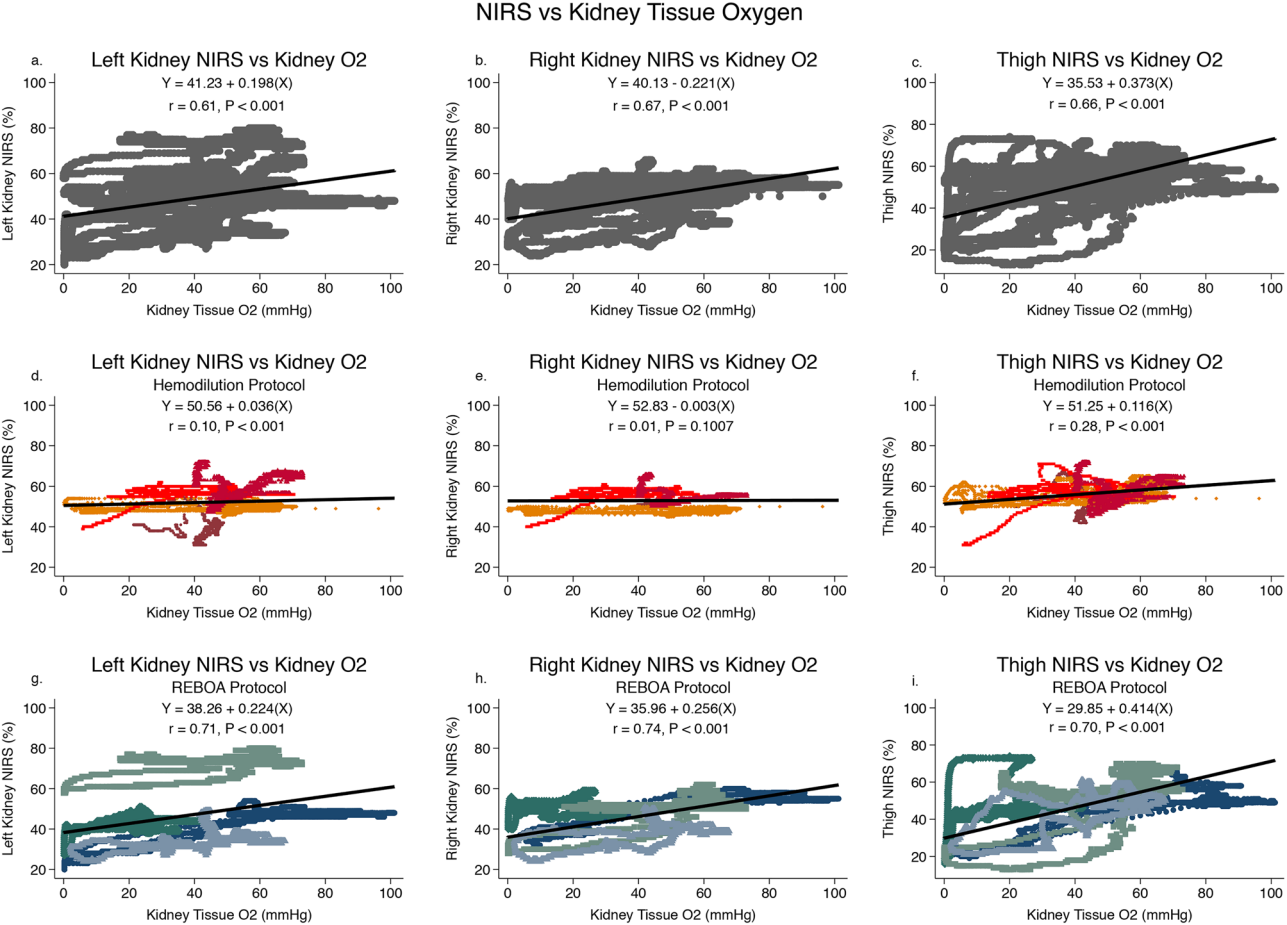
Left kidney, right kidney, and thigh near infra-red spectroscopy (NIRS) are compared to direct kidney tissue oxygen measurements for all 8 animals (grey symbols), the 4 animals undergoing the Hemodilution protocol (maroon, orange, bright red, dark red) and the 4 animals undergoing the REBOA protocol (green, teal, light blue, dark blue). In graphs d– i, each individual animal is represented by a different color. The mixed effect linear regression model is plotted as a solid black line and the equation of that line is reported. The within group correlation coefficient is reported as “r”. NIRS data were sampled every 2 s and kidney tissue oxygen was sampled every second.

**Figure 5:**
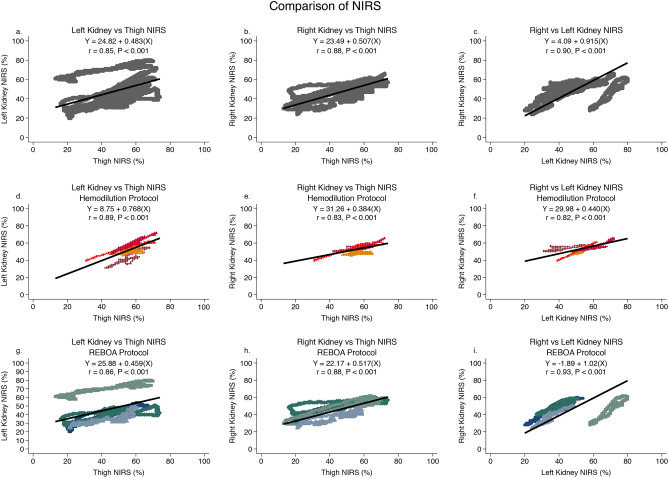
Left kidney, right kidney, and thigh near infra-red spectroscopy (NIRS) are compared to each other for all 8 animals (grey symbols), the 4 animals undergoing the Hemodilution protocol (maroon, orange, bright red, dark red) and the 4 animals undergoing the REBOA protocol (green, teal, light blue, dark blue). In graphs d–i, each individual animal is represented by a different color. The mixed effect linear regression model is plotted as a solid black line and the equation of that line is reported. The within group correlation coefficient is reported as “r”. NIRS data were sampled every 2 s and kidney tissue oxygen was sampled every second.

Figure 4 shows the comparison of kidney NIRS to thigh NIRS measurements when data are pooled from all 8 animals. There was a strong correlation between both kidney NIRS signals and thigh NIRS measurements (*r* = 0.85 *p* < 0.001;*r* = 0.88 *p* < 0.001;for left kidney vs thigh and right kidney vs thigh respectively). There was also a strong correlation between left and right kidney NIRS (*r* = 0.90 *p* < 0.001). These relationships were maintained regardless of the resuscitation protocol (Hemodillution group *r* = 0.89 *p* < 0.001; *r* = 0.83 *p* < 0.001; for left kidney vs thigh and right kidney vs thigh respectively and REBOA group *r* = 0.86 *p* < 0.001; *r* = 0.88 *p* < 0.001; for left vs thigh and right vs thigh respectively) Left and right kidney NIRS were also highly correlated regardless of protocol (Hemodillution group *r* = 0.82 *p* < 0.001 and REBOA group *r* = 0.93 *p* < 0.001) (Fig. 5).

Supplemental Fig. 2 shows the Bland–Altman analysis for the comparison of NIRS measurements with each other. The mean difference and limits of agreement of left kidney NIRS vs thigh NIRS and right kidney NIRS vs thigh NIRS were 0.02% (− 20.42, 20.45) and − 1.24% (− 15.26, 12.79) respectively. The mean difference and limits of agreement when comparing left and right kidney NIRS were 1.25% (− 17.64, 20.14).

## Discussion

In our animal model of hemorrhage, hemodilution, and REBOA, we showed a moderate correlation between kidney tissue oxygen and regional NIRS oxygen saturations but this relationship was only apparent during the extreme hemodynamic changes that occurred with balloon occlusion of the aorta. The correlation was poor or insignificant during more subtle changes, such as hemorrhage and hemodilution. Moreover, in all animals, the strongest correlations found were between the NIRS measurements themselves (kidney vs thigh and left vs right kidney) regardless of the experimental protocol. Taken together, these data suggest that regional NIRS measurements made with sensors carefully placed over the kidney with ultrasound were more closely related to thigh NIRS than invasive kidney oxygen concentration, even when the surface of the kidney was between 1.5 and 2 cm from the skin.

Components of biological tissues, including hemoglobin and water, can absorb near-infrared light. Hemoglobin, for instance, exhibits strong absorption peaks within the near-infrared range^25^. This allows NIRS sensors to assess tissue capillary hemoglobin concentration and oxygenation saturation. Light scattering occurs when near-infrared photons interact with cellular and subcellular structures, causing deviations in the photons' trajectories that are challenging to predict and lead to variations in the light's penetration depth. As a result, the measured signal is a weighted average of oxygen levels in the various tissues beneath the skin's surface.

Absorption and scattering of light by tissues also limits the maximum penetration depth of the near-infrared light employed by NIRS sensors. Moreover, the NIRS measurement is made through diffuse reflectance rather than direct transmission, further limiting the depth of tissues that can be measured. The manufacturer of the NIRS sensor used in this study states that the maximum depth of penetration is 2.5 centimeters^26^. The depth of penetration of NIRS measurements, however, likely depends on the optical properties of the tissue being measured, the size and type of sensor used, the source-detector separation, and the specific algorithm used^7,27^. Patil et al. modeled NIRS experimentally and found a nonlinear relationship between source-detector separation and depth of penetration such that a source-detector separation of 20 mm resulted in a depth of penetration of 10 mm but a source-detector separation of 40 mm resulted in a depth of penetration of 15mm^7^. The sensors we used had one emitter and two detectors with source-detectors distances of 15 mm and 50 mm. If the results of Patil’s experimental model can be applied to our sensors and the optical properties of subcutaneous and kidney tissue, then extrapolating from their data, the range of penetration for our study should have been between 8 and 17 mm.

In our animal model, the surface of the kidney was between 1.5 and 2 cm (15–20 mm) below the skin. The weighted average of tissue oxygen measured by NIRS, therefore, was likely to be substantially more affected by subcutaneous tissue than kidney tissue. In addition, the anatomy of the porcine and adult human kidney is such that the medullary tissue is 1–2 cm from the surface of the kidney. This makes it even less likely that any significant component of the weighted average of tissue oxygen measured by NIRS was due to medullary tissue oxygenation and thus may explain why the NIRS measurements we obtained over the kidney were more closely related to thigh NIRS than direct medullary tissue oxygen concentration. Any correlation we found likely represents a relationship of kidney perfusion and global perfusion determinants such as mean arterial pressure and cardiac output.

Clinical studies placing NIRS sensors on the skin over the kidney in adult cardiac surgery patients have shown an association of NIRS oxygenation measurements and the subsequent development of AKI^17,18^. In these studies patients were excluded if the surface of the kidney was > 4 cm from the skin by ultrasound. The distance of the kidney from the skin in these adult human studies was therefore greater than in our animal model and the contribution of medullary tissue oxygen to the weighted average of their NIRS measurements was likely even less than in our model. The relationship these studies found between regional tissue oxygen measured using NIRS and outcome therefore may be related to the ability of NIRS to monitor global perfusion and capillary tissue oxygenation of the subcutaneous tissue in these patients. Indeed, a study in adult cardiac surgery patients showed that NIRS sensors placed over the thigh predicted subsequent AKI with receiver operator characteristic analysis suggesting an optimal cut-off of 67% and an area under the curve of 0.84^28^. It is therefore possible that regional oxygen saturation measurements of subcutaneous tissue are good predictors of global malperfusion, whether they are placed over the subcutaneous tissue of the kidney or the thigh.

Several studies have compared NIRS measurements to either direct tissue oxygen measurements or venous oxygen saturation. Skowno et al. compared transcutaneous hepatic tissue oxygen saturation using NIRS to direct hepatic tissue oxygen measurements in juvenile pigs and found that in animals weighing 15-20 kg, hepatic artery clamping was associated with an 84% decrease in direct hepatic oxygen concentration compared to a 6% decrease in NIRS measurements^29^. In 37 children undergoing cardiac catheterization, Ortmann et al. found that measurements from NIRS sensors placed over the flank in children < 10 kg were strongly correlated with renal vein oxygen saturation (*r* = 0.821; *p* = 0.002) but in children > 10 kg there was no significant correlation^30^. In contrast, Tholen et al. found a statistically significant correlation between kidney NIRS and renal venous oxygen saturation in 13 adult cardiac surgery patients with a continuous cardiac output pulmonary catheter placed in the renal vein^19^. The correlation coefficients found in this adult study, however, ranged from 0.51 to 0.75 and so were similar to the results we found between NIRS and direct kidney tissue oxygen measurements regardless of whether NIRS sensors were placed over the kidney or over the thigh. If it is true that thigh NIRS monitoring may be as effective at predicting poor perfusion and AKI as placing sensors over the kidney, then NIRS monitoring could be used in adult patients regardless of their BMI. Future studies are needed to ascertain whether or not changes in clinical management based on these subcutaneous tissue oxygen measurements can improve kidney oxygenation or reduce the incidence of AKI in critically ill patients.

We found the most robust relationship between NIRS oxygen monitoring (whether over the kidney or the thigh) and kidney tissue oxygen was during aortic occlusion and release with REBOA. This relationship was likely because of the extreme decrease in blood flow to all regions distal to the aortic balloon. REBOA creates precise and reproducible aortic occlusion. While it is a useful tool to evaluate renal tissue perfusion across a wide range of renal blood flows, it is also a technique with direct clinical applicability. Many studies have established that REBOA is best performed by partially deflating the balloon after a brief period of full occlusion^31–33^. Partial REBOA prevents both total lower-body ischemia and the deleteriously high blood pressure in the upper extremity that occur with complete REBOA. A porcine hemorrhage and REBOA study demonstrated a reduction in serum lactate concentration and less duodenal necrosis with 50% occlusion compared to complete REBOA^34^. Our data suggest that thigh NIRS might be a noninvasive and clinically available surrogate for lower body perfusion during REBOA. Future studies will evaluate the efficacy of titrating partial REBOA to maximize upper and lower body oxygen delivery using NIRS guidance.

One limitation of our study is that we only compared NIRS oxygen saturations to medullary kidney tissue oxygen concentration. While the surface of the kidney was only 1.5–2 cm from the skin in all of our animals, the medulla was often 1–2 cm deeper than this. Therefore, if we had measured kidney tissue oxygen in the more superficial cortex tissue, we might have found a closer relationship with NIRS oxygen saturation measurements from sensors placed immediately above the kidney. We measured medullary oxygen because this kidney region has the lowest oxygen reserve and is thought to be responsible for most AKI caused by hypoperfusion^35,36^. A second limitation relates to the methodology of comparing two different technologies for measuring tissue oxygenation. Measurements using the same technology are subject to similar errors and therefore are likely to show a higher correlation compared to measurements from differing technologies. This may have contributed to the closer correlation when the various NIRS measurements were compared to each other rather than when each NIRS measurement was compared to invasive kidney oxygen concentration. A third limitation of the study is that we had such an abundance of data (collected every 1–2 s for hours in each animal) that statistical significance was easily obtained even when the correlation between variables was poor. This large dataset also precluded a statistical comparison between correlation coefficients because all the relationships were statistically significantly different even when the correlation coefficients differed by 0.01 (a clinically insignificant difference). In addition, the variability in NIRS measurements between animals was sometimes as large or larger than the variation over time within an individual animal. This large variability in NIRS measurements between animals might be partially explained by the two very different resuscitation protocols. Subtle differences in NIRS sensor placement might have also been a factor. This inter-subject variability was accounted for statistically by using a “within-subject” correlation coefficient that removes the variation between subjects, and by using a mixed effect linear regression which accounts for the lack of independence among repeated measures when making between-subjects comparisons. Nonetheless, the correlations we found between NIRS measurements and invasive kidney oxygen concentration, at best suggest that NIRS could be used as a trend monitor suggesting changes in kidney oxygen rather than a precise indicator of kidney oxygen concentration. Finally, our study only assessed the correlation between invasive kidney oxygen measurements and noninvasive NIRS monitoring. We did not evaluate the ability of noninvasive NIRS monitoring to predict actual kidney injury, nor did we ascertain whether hemodynamic management based on NIRS monitoring could prevent AKI. Even if NIRS placed over the kidney is only a subcutaneous tissue monitor, skin and muscle are highly vascular organs, and maintaining their perfusion may ensure the perfusion of other vital organs, such as the kidney. Future work should focus on whether an intervention based on regional oxygen monitoring with NIRS can reduce the incidence or severity of AKI in critically ill and injured patients.

## Conclusion

In our porcine model of hemorrhage, hemodilution, and resuscitation, NIRS measurements from sensors placed on the skin over both the kidney and the thigh showed a moderate correlation with invasive kidney oxygen concentrations during extreme hemodynamic changes such as aortic occlusion. During the more mild hemodynamic changes of hemodilution the correlation was poor or insignificant. Furthermore, kidney NIRS and thigh NIRS measurements were closely related suggesting that both might reflect subcutaneous tissue oxygenation even when the surface of the kidney is 1.5–2 cm from the skin. These data shed light on other human studies of kidney NIRS which have shown association with renal vein oxygen saturation and outcome. Further studies are needed to determine whether this subcutaneous tissue oxygen monitoring with NIRS can be used to guide adult hemodynamic management or the implementation of partial REBOA.

### Supplementary Information

Supplementary Figure 1.

Supplementary Figure 2.

## Data Availability

The datasets during and/or analyzed during the current study are available from the corresponding author upon reasonable request.

## References

[1] Biesterveld BE, Siddiqui AZ, O'Connell RL, Remmer H, Williams AM, Shamshad A, Smith WM, Kemp MT, Wakam GK, Alam HB (2021). Valproic acid protects against acute kidney injury in hemorrhage and trauma. J. Surg. Res..

[2] Rhee P, Joseph B, Pandit V, Aziz H, Vercruysse G, Kulvatunyou N, Friese RS (2014). Increasing trauma deaths in the United States. Ann. Surg..

[3] Kauvar DS, Lefering R, Wade CE (2006). Impact of hemorrhage on trauma outcome: An overview of epidemiology, clinical presentations, and therapeutic considerations. J. Trauma..

[4] Harrois A, Soyer B, Gauss T, Hamada S, Raux M, Duranteau J, Traumabase G (2018). Prevalence and risk factors for acute kidney injury among trauma patients: a multicenter cohort study. Crit Care..

[5] Booth EA, Dukatz C, Ausman J, Wider M (2010). Cerebral and somatic venous oximetry in adults and infants. Surg. Neurol. Int..

[6] Benni PB, MacLeod D, Ikeda K, Lin HM (2018). A validation method for near-infrared spectroscopy based tissue oximeters for cerebral and somatic tissue oxygen saturation measurements. J. Clin. Monit. Comput..

[7] Patil AV, Safaie J, Moghaddam HA, Wallois F, Grebe R (2011). Experimental investigation of NIRS spatial sensitivity. Biomed. Opt Express..

[8] Nagdyman N, Ewert P, Peters B, Miera O, Fleck T, Berger F (2008). Comparison of different near-infrared spectroscopic cerebral oxygenation indices with central venous and jugular venous oxygenation saturation in children. Paediatr. Anaesth..

[9] Bruckner M, Wolfsberger CH, Dempsey EM, Liem KD, Lemmers P, Alderliesten T, Alarcon A, Mintzer J, de Boode WP, Schmolzer GM, Pichler G, Spectroscopy ESIGNI (2021). Normal regional tissue oxygen saturation in neonates: a systematic qualitative review. . Pediatr. Res..

[10] Gil-Anton J, Redondo S, Garcia Urabayen D, Nieto Faza M, Sanz I, Pilar J (2015). Combined cerebral and renal near-infrared spectroscopy after congenital heart surgery. Pediatr. Cardiol..

[11] Gist KM, Kaufman J, da Cruz EM, Friesen RH, Crumback SL, Linders M, Edelstein C, Altmann C, Palmer C, Jalal D, Faubel S (2016). A decline in intraoperative renal near-infrared spectroscopy is associated with adverse outcomes in children following cardiac surgery. Pediatr. Crit. Care Med..

[12] Hanson SJ, Berens RJ, Havens PL, Kim MK, Hoffman GM (2009). Effect of volume resuscitation on regional perfusion in dehydrated pediatric patients as measured by two-site near-infrared spectroscopy. Pediatr. Emerg. Care..

[13] Marin T, Williams BL (2021). Renal oxygenation measured by near-infrared spectroscopy in neonates. Adv. Neonatal. Care..

[14] Suzuki S, Takasaki S, Ozaki T, Kobayashi Y. Tissue oxygenation monitor using NIR spatially resolved spectroscopy. Society of Photo-Optical Instrumentation Engineers (SPIE), Optical Tomography and Spectroscopy of Tissue III. pp. 582–92. (1999)

[15] Quaresima V, Ferrari M (2016). Functional near-infrared spectroscopy (fNIRS) for assessing cerebral cortex function during human behavior in natural/social situations: A concise review. Organ. Res. Methods.

[16] Johnson A, Roskosky M, Freedman B, Shuler MS (2015). Depth penetration of near infrared spectroscopy in the obese. J. Trauma. Treat..

[17] Choi DK, Kim WJ, Chin JH, Lee EH, Don Hahm K, Yeon Sim J, Cheol CI (2014). Intraoperative renal regional oxygen desaturation can be a predictor for acute kidney injury after cardiac surgery. J. Cardiothorac. Vasc. Anesth..

[18] Ortega-Loubon C, Fernandez-Molina M, Fierro I, Jorge-Monjas P, Carrascal Y, Gomez-Herreras JI, Tamayo E (2019). Postoperative kidney oxygen saturation as a novel marker for acute kidney injury after adult cardiac surgery. J. Thorac. Cardiovasc. Surg..

[19] Tholen M, Ricksten SE, Lannemyr L (2020). Renal near-infrared spectroscopy for assessment of renal oxygenation in adults undergoing cardiac surgery: a method validation study. J. Cardiothorac. Vasc. Anesth..

[20] Lofgren LR, Hoareau GL, Kuck K, Silverton NA (2022). Noninvasive and invasive renal hypoxia monitoring in a porcine model of hemorrhagic shock. J. Vis. Exp..

[21] Hoareau GL, Williams TK, Davidson AJ, Russo RM, Ferencz SE, Neff LP, Grayson JK, Stewart IJ, Johnson MA (2019). Endocrine effects of simulated complete and partial aortic occlusion in a swine model of hemorrhagic shock. Mil Med..

[22] Abid M, Neff LP, Russo RM, Hoareau G, Williams TK, Grayson JK, DuBose JJ, Lendrum R, Johnson MA (2020). Reperfusion repercussions: A review of the metabolic derangements following resuscitative endovascular balloon occlusion of the aorta. J. Trauma. Acute. Care Surg..

[23] Patel NTP, Gaffley M, Leblanc MJR, Lane MR, Hoareau GL, Johnson MA, Jordan JE, Neff LP, Williams TK (2022). Endovascular perfusion augmentation after resuscitative endovascular balloon occlusion of the aorta improves renal perfusion and decreases vasopressors. J. Surg. Res..

[24] Bland JM, Altman DG (1999). Measuring agreement in method comparison studies. Stat. Methods Med. Res..

[25] Ayaz H, Izzetoglu M, Izzetoglu K, Onaral B, Ben DB (2019). Early diagnosis of traumatic intracranial hematomas. J. Biomed. Opt..

[26] [12/04/2023]Edwards ForeSight Manufactorer’s Website]. Available from: https://www.edwards.com/healthcare-professionals/products-services/tissue-oximetry/foresight.

[27] Steppan J, Hogue CW (2014). Cerebral and tissue oximetry. Best Pract. Res. Clin. Anaesthesiol..

[28] Evans RG, Smith DW, Lee CJ, Ngo JP, Gardiner BS (2020). What makes the kidney susceptible to hypoxia?. Anat. Rec. Hoboken..

[29] Sakaki K, Kitamura T, Kohira S, Torii S, Mishima T, Hanayama N, Kobayashi K, Ohkubo H, Miyaji K (2020). Regional thigh tissue oxygen saturation during cardiopulmonary bypass predicts acute kidney injury after cardiac surgery. J. Artif. Organs..

[30] Skowno JJ, Karpelowsky JS, Watts NR, Little DG (2016). Can transcutaneous near infrared spectroscopy detect severe hepatic ischemia: a juvenile porcine model. Paediatr. Anaesth..

[31] Ortmann LA, Fontenot EE, Seib PM, Eble BK, Brown R, Bhutta AT (2011). Use of near-infrared spectroscopy for estimation of renal oxygenation in children with heart disease. Pediatr. Cardiol..

[32] Hoareau GL, Beyer CA, Caples CA, Spruce MW, Kevin Grayson J, Neff LP, Williams TK, Johnson MA (2020). Automated partial versus complete resuscitative endovascular balloon occlusion of the aorta for the management of hemorrhagic shock in a pig model of polytrauma: A randomized controlled pilot study. Mil. Med..

[33] Williams TK, Tibbits EM, Hoareau GL, Simon MA, Davidson AJ, DeSoucy ES, Faulconer ER, Grayson JK, Neff LP, Johnson MA (2018). Endovascular variable aortic control (EVAC) versus resuscitative endovascular balloon occlusion of the aorta (REBOA) in a swine model of hemorrhage and ischemia reperfusion injury. J. Trauma. Acute Care Surg..

[34] Johnson MA, Tibbits EM, Hoareau GL, Simon MA, Davidson AJ, DeSoucy ES, Faulconer ER, Grayson JK, Neff LP, Williams TK (2019). Endovascular perfusion augmentation for critical care: Partial aortic occlusion for treatment of severe ischemia-reperfusion shock. Shock..

[35] Russo RM, Neff LP, Lamb CM, Cannon JW, Galante JM, Clement NF, Grayson JK, Williams TK (2016). Partial resuscitative endovascular balloon occlusion of the aorta in swine model of hemorrhagic shock. J. Am. Coll. Surg..

[36] Evans RG, Ince C, Joles JA, Smith DW, May CN, O'Connor PM, Gardiner BS (2013). Haemodynamic influences on kidney oxygenation: clinical implications of integrative physiology. Clin. Exp. Pharmacol. Physiol..

