# Supplementary material for: Near-infrared spectroscopy for kidney oxygen monitoring in a porcine model of hemorrhagic shock, hemodilution, and REBOA
Source: Sci Rep. 2024 Feb 1;14:2646. doi: 10.1038/s41598-024-51886-y (PMC10834443; doi:10.1038/s41598-024-51886-y)

Supplemental Figure 1: Pre-specified algorithm for the resuscitation of animals after the return of shed blood and/or the completion of resuscitative endovascular balloon aortic occlusion (REBOA). Isotonic crystalloid and norepinephrine are used to maintain mean arterial pressure (MAP) > 65mmHg. The decision to use an intravenous bolus of isotonic crystalloid vs norepinephrine is based on central venous pressure (CVP) measurement.

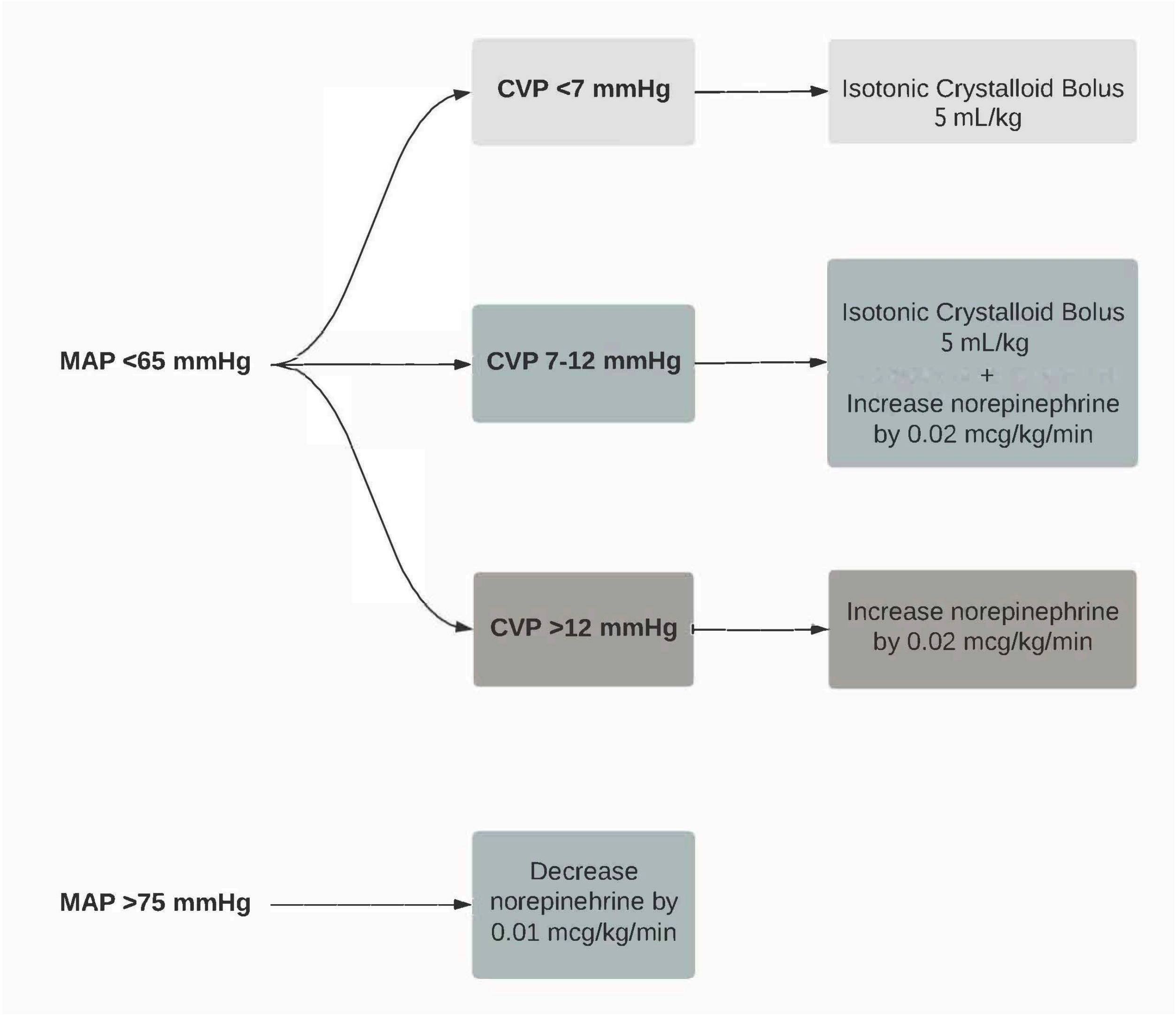

Supplement: Supplementary file 1 — Supplementary Figure 1. [file 41598_2024_51886_MOESM1_ESM.pdf]
