# Supplementary material for: Near-infrared spectroscopy for kidney oxygen monitoring in a porcine model of hemorrhagic shock, hemodilution, and REBOA
Source: Sci Rep. 2024 Feb 1;14:2646. doi: 10.1038/s41598-024-51886-y (PMC10834443; doi:10.1038/s41598-024-51886-y)

Supplemental Figure 2: Bland-Altman analysis for the comparison of near infra-red spectroscopy (NIRS) measurements. The mean difference is represented by a black solid line and the 95% limits of agreement are represented by red dashed lines.

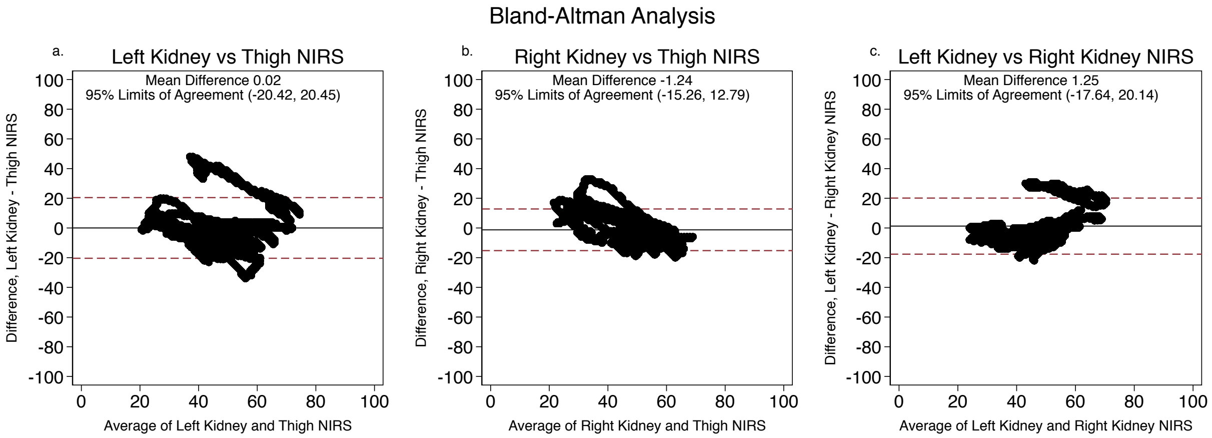

Supplement: Supplementary file 2 — Supplementary Figure 2. [file 41598_2024_51886_MOESM2_ESM.pdf]
